# Cognitive frailty and cardiometabolic risk in middle-aged and older adults: evidence from the UK and China

**DOI:** 10.1007/s40520-025-03179-1

**Published:** 2025-09-04

**Authors:** Haiyang Yan, Jingjing Lang, Chengfeng Li, Samaneh Eftekhariranjbar, Guoyan Jiang, Jing Lei, Lixin Sun, Carlos J. Toro-Huamanchumo, Zhongyang Guan

**Affiliations:** 1 Yancheng Municipal Center for Disease Control and Prevention, Yancheng, Jiangsu China; 2 Children’s Hospital of Soochow University, Suzhou, Jiangsu China; 3 Nutrition and Health Innovation Research Institute, School of Medical and Health Sciences, Edith Cowan University, Western Australia, Australia; 4 University “G. D’Annunzio” of Chieti-Pescara, Chieti, Italy; 5 Sapienza University of Rome, Rome, Italy; 6 Curtin Medical School, Faculty of Health Sciences, Curtin University, Perth, Western Australia Australia; 7 Center for Health Policy and Development Research, Jiangxi Science and Technology Normal University, Nanchang, Jiangxi China; 8 OBEMET Center for Obesity and Metabolic Health, Lima, Peru; 9 Research Unit for Health Evidence Generation and Synthesis, Universidad San Ignacio de Loyola, Lima, Peru; 10 School of Population Health, Faculty of Health Sciences, Curtin University, Perth, Western Australia Australia; 11 Dementia Centre of Excellence, enAble Institute, Curtin University, Perth, Western Australia Australia

**Keywords:** Frailty, Cognitive impairment, Cognitive frailty, Cardiometabolic disease

## Abstract

**Background:**

Cognitive frailty, a novel construct integrating cognitive and physical deficits, is increasingly recognized in aging research.

**Aims:**

This study aimed to examine the associations between cognitive frailty and cardiometabolic risk in two nationally representative cohorts from China and the United Kingdom.

**Methods:**

We analyzed data from 7,628 participants in the China Health and Retirement Longitudinal Study (CHARLS) and 4,703 participants from the English Longitudinal Study of Ageing (ELSA), all aged ≥ 50 years. Frailty was assessed using the frailty index (FI) in the main analysis. Cox proportional hazards models were applied to estimate hazard ratios (HRs) for incident cardiometabolic diseases (CMDs), cardiovascular diseases (CVDs), and diabetes. Subgroup and interaction analyses were performed to examine effect modification. Restricted cubic spline (RCS) models were used to assess the shape of the association between FI and cardiometabolic risk. Sensitivity analyses employed competing risk models and the physical frailty phenotype (PFP) as an alternative frailty measure.

**Results:**

Cognitive frailty was associated with higher risks of CMDs (HR 1.58, 95% CI 1.39–1.79), CVDs (HR 1.64, 95% CI 1.42–1.89), and diabetes (HR 1.39, 95% CI 1.11–1.75). Cognitive impairment alone showed no significant association with these outcomes in the main analysis. Dose–response associations were significant between the FI and CMDs and CVDs among individuals with and without cognitive impairment. Results were consistent across cohorts and robust in sensitivity analyses.

**Conclusions:**

Cognitive frailty is a consistent predictor of cardiometabolic risk across distinct populations, supporting integrated screening and prevention strategies targeting both cognitive and physical deficits in aging populations.

**Supplementary Information:**

The online version contains supplementary material available at 10.1007/s40520-025-03179-1.

## Introduction

As the global population ages at an unprecedented rate, age-related health conditions have become increasingly prevalent. Among these, frailty and cognitive impairment are two of the most prevalent geriatric syndromes [1–3]. Globally, an estimated 12% of individuals aged 50 years and older are currently classified as frail [1], while the prevalence of mild cognitive impairment in this age group is approximately 20% [3]. Frailty is a biological syndrome characterized by reduced physiological reserve and diminished resistance to stressors, resulting from cumulative declines across multiple physiological systems [4, 5]. Cognitive impairment refers to a decline in intellectual functions, including thinking, memory, reasoning, and planning [2]. Given the potential bidirectional relationship and shared pathophysiological mechanisms—such as chronic inflammation, oxidative stress, and mitochondrial dysfunction—these two conditions have been conceptualized collectively as cognitive frailty, defined as the concurrent presence of both conditions in older adults without dementia [6, 7].

Both cognitive impairment and frailty are key markers of the aging process and are associated with a range of adverse outcomes [8–10], including disability, hospitalization, mortality, and cardiometabolic diseases (CMDs) [11, 12]. However, most existing research has focused on either frailty or cognitive impairment alone, rather than their co-occurrence. For instance, a Mendelian randomization study by Zhu et al. demonstrated bidirectional genetic associations between frailty and CMDs including coronary artery disease, stroke, and type 2 diabetes [13]. Similarly, a recent meta-analysis reported a bidirectional association between frailty and diabetes [14]. In contrast, several previous studies suggested that the synergistic effect of co-existing frailty and cognitive impairment is associated with a heightened risk of adverse health outcomes [15, 16]. Specifically, a longitudinal study across 17 countries found that individuals with cognitive frailty (subdistribution hazard ratio [SHR] 2.34, 95% CI 2.01–2.72) had greater risk of mortality than those with either frailty (SHR 1.83, 95% CI 1.72–1.95) or cognitive impairment (SHR 1.36, 95% CI 1.25–1.48) alone [16]. Nonetheless, whether cognitive frailty confers greater risk of CMDs—a leading cause of death worldwide—than either condition in isolation remains unclear [12]. Furthermore, the underlying mechanisms linking cognitive frailty with the development and progression of CMDs have yet to be fully elucidated.

Our study utilized data from two nationally representative cohorts of middle-aged and older adults in the UK (English Longitudinal Study of Ageing [ELSA]) and China (China Health and Retirement Longitudinal Study [CHARLS]) to examine the associations of cognitive frailty with the risk of incident CMDs [17, 18]. Given the differing sociocultural and healthcare contexts in these two countries, this cross-national approach may help to identify context-specific patterns and inform globally relevant strategies for addressing cardiometabolic vulnerability associated with frailty and cognitive impairment in diverse aging populations.

## Methods

### Study population

Detailed study designs of the CHARLS and ELSA can be found in Supplementary Information. For the current study, participants from these two cohorts were excluded if they met any of the following criteria: (1) were below 50 years of age at baseline; (2) had prevalent CMDs, dementia, or Parkinson’s disease at baseline; (3) lacked follow-up data on cardiometabolic outcomes of interest; or (4) had insufficient data to assess frailty status and cognitive function. Participant selection procedures are presented in Fig. **S1**.

### Assessment of frailty

Frailty was primarily assessed using the frailty index (FI), given its ability to capture a broad range of health deficits including functional, psychological, and comorbid conditions. The physical frailty phenotype (PFP) was additionally used in sensitivity analyses to evaluate the robustness of findings [19, 20]. To harmonize frailty assessments across the CHARLS and ELSA datasets, 26 items were selected for FI construction, comprising self-reported health status, chronic diseases (excluding CMDs), physical function, and psychological conditions (Table **S1**). The 26-item FI was developed in accordance with standard guidelines and based on prior studies using the CHARLS and ELSA cohorts [16, 19, 21]. For all variables included in the FI, a score of 0 indicated the absence of a deficit, and a score of 1 indicated the presence of a deficit. The FI score was calculated as the ratio of the number of deficits present to the total number of deficits considered, with a higher score indicating greater frailty. Based on previous studies [21, 22], FI scores were categorized into three groups: robust (FI score ≤ 0.10), prefrail (FI score > 0.10 and < 0.25), and frail (FI score ≥ 0.25). Participants with more than 20% missing data across FI items (i.e., more than 5 items) were excluded (Fig. **S1**) [23]. The details of the PFP approach can be found in Supplementary Information.

### Assessment of cognitive impairment

For CHARLS participants, cognitive impairment was assessed using a combination of three tests: specifically, the Telephone Interview for Cognitive Status (TICS-10), a word recall test, and a figure drawing test. The composite score of these three tests ranges from 0 to 21, with higher scores indicating better cognitive function [24]. For ELSA participants, cognitive impairment was assessed based on three tests: the word recall test (immediate and delayed recall, with a total score of 20 [10 points each]), the date naming test (maximum score of 4), and the verbal fluency test. In the verbal fluency test, participants were asked to name as many animals as possible within 60 s, and the number of animals named was recorded as the test score [25]. Participants scoring more than one standard deviation (SD) below age-appropriate norms were classified as cognitively impaired. Those scoring within one standard deviation of the norm or above were considered cognitively normal [26]. The procedures for these cognitive tests in the two cohorts are described in detail elsewhere [24, 25].

### Assessment of cognitive frailty

Participants were categorized according to their frailty and cognitive status into the following groups: normal (non-frail and normal cognition), frailty only, cognitive impairment only, and cognitive frailty (co-occurrence of cognitive impairment and frailty).

### Ascertainment of outcomes and endpoints

The primary outcome was incident major CMDs, defined as the occurrence of either major cardiovascular diseases (CVDs; including heart diseases and stroke) or diabetes. Incident major CVDs and diabetes were also examined separately as secondary outcomes. In each wave of CHARLS and ELSA, participants were asked whether a doctor had informed them of a diagnosis of diabetes, heart diseases (including angina, heart attack, congestive heart failure, and other heart problems), or stroke. Participants reporting a diagnosis of heart disease or stroke were classified as having incident CVDs, and those reporting a diagnosis of diabetes were classified as having incident diabetes. Follow-up continued until the first occurrence of a major CMD, death, or the end of the follow-up period, whichever came first.

### Covariates

The covariates included age (in years), sex (female or male), study region (China or the UK), marital status, education level, smoking status, alcohol consumption, and physician-diagnosed hypertension [16]. To ensure consistency between CHARLS and ELSA, marital status was dichotomized as either married/partnered or other (including separated, divorced, unmarried, or widowed). Education level was classified into two categories: less than high school and high school or above. Smoking status was grouped as current smokers, former smokers, or never smokers. Alcohol consumption was categorized as never drinkers and ever drinkers. Physician-diagnosed hypertension was included as a covariate but was not considered part of the major CMDs definition.

### Statistical analysis

Descriptive characteristics were summarized across the four frailty and cognitive groups. Baseline characteristics were also compared between participants who completed follow-up and those lost to follow-up to assess potential selection bias. Cox proportional hazards regression models were used to investigate the associations of cognitive frailty with the risk of three cardiometabolic outcomes. The proportional hazards assumption was assessed using Schoenfeld residuals, with no violations detected. In the main analysis, frailty was assessed using the FI, and the normal group served as the reference category. For the primary outcome (major CMDs) and two secondary outcomes (major CVDs and diabetes), hazard ratios (HRs) and 95% confidence intervals (CIs) were estimated using two models: Model 1 adjusted for age and sex; Model 2 further adjusted for study region (China or the UK), marital status, education level, smoking status, alcohol consumption, and hypertension. To assess potential synergistic effects, risks among participants with cognitive frailty were compared with those in participants with frailty alone or cognitive impairment alone. Subgroup analyses were conducted by study region (China vs. UK) using separate Cox models based on Model 2 (excluding the stratification variable). Region-specific adjusted survival curves were generated to illustrate time-to-event differences across cognitive–frailty groups. Further stratified analyses were conducted within each cohort by age group (50–65 vs. >65 years) and by sex, with stratification variables excluded from the respective models. Multiplicative interaction terms between cognitive–frailty status and age group or sex were included to evaluate effect modification.

To further examine the association between frailty and cardiometabolic outcomes across different cognitive states, we conducted restricted cubic spline (RCS) regression models stratified by cognitive status (with vs. without cognitive impairment). In each subgroup, FI was modeled as a continuous exposure using Cox proportional hazards models (Model 2), with FI = 0.25 set as the reference value. To evaluate the robustness of our findings, two sets of sensitivity analyses were performed. First, all primary and region-stratified analyses were re-estimated using Fine*-*Gray subdistribution hazard models to account for death as a competing risk [27]. Second, the associations were re-evaluated using the PFP as an alternative frailty measure [20], applying the same modeling strategy and covariate adjustments as in the main analyses.

All statistical analyses were performed using Stata version 18.0 (StataCorp LLC, College Station, TX, USA) and R version 4.4.1 (R Foundation for Statistical Computing, Vienna, Austria). A *P*-value of less than 0.05 was considered statistically significant.

## Results

### Participant selection and characteristics

A total of 7,628 participants from CHARLS and 4,703 from ELSA were included in the final analytical sample (Fig. **S1**). Baseline characteristics of participants stratified by cognitive and frailty status are presented in Table 1. In both CHARLS and ELSA, statistically significant differences were observed across the four groups for all sociodemographic and health-related variables (*P* < 0.0001). Individuals with cognitive frailty tended to be older, more likely to be female, less educated, and less likely to be married or partnered. As shown in Table S2 participants who were lost to follow-up differed significantly from those retained across several baseline characteristics, including age, education, and marital status. Baseline characteristics of participants who were excluded due to insufficient data for frailty assessment are presented in Table S3.

**Table 1:** Baseline characteristics of the final analytical sample by cognitive impairment and frailty status

	CHARLS (*n* = 7,628)						ELSA (*n* = 4,703)				
	Normal (*n* = 4,948)	Frailty only (*n* = 1,194)	Cognitive impairment only (*n* = 928)	Cognitive frailty (*n* = 558)	*P* value		Normal (*n* = 3,181)	Frailty only (*n* = 455)	Cognitive impairment only (*n* = 831)	Cognitive frailty (*n* = 236)	*P* value
Age (years), mean (SD)	60.6 (7.5)	63.3 (8.1)	61.4 (8.2)	65.0 (9.3)	< 0.0001		62.8 (7.8)	65.1 (9.1)	69.2 (9.7)	71.3 (10.5)	< 0.0001
Sex, n (%)					< 0.0001						< 0.0001
Female	1,972 (39.9)	663 (55.5)	663 (71.4)	444 (79.6)			1,748 (55.0)	333 (73.2)	457 (55.0)	167 (70.8)	
Male	2,976 (60.2)	531 (44.5)	265 (28.6)	114 (20.4)			1,433 (45.1)	122 (26.8)	374 (45.0)	69 (29.2)	
Education, n (%)					< 0.0001						< 0.0001
High school not completed	4,304 (87.0)	1,137 (95.2)	916 (98.7)	557 (99.8)			941 (32.1)	199 (49.8)	489 (64.2)	162 (76.8)	
High school or above	644 (13.0)	57 (4.8)	12 (1.3)	1 (0.2)			1,992 (67.9)	201 (50.3)	273 (35.8)	49 (23.2)	
Marital status, n (%)					< 0.0001						< 0.0001
Married or partnered	4,219 (85.3)	942 (78.9)	743 (80.1)	396 (71.0)			2,361 (74.2)	260 (57.1)	504 (60.7)	113 (47.9)	
Others	729 (14.7)	252 (21.1)	185 (19.9)	162 (29.0)			829 (25.8)	195 (42.9)	326 (39.3)	123 (52.1)	
Alcohol consumption, n (%)					< 0.0001						< 0.0001
Never drinkers	2,656 (53.7)	738 (61.8)	664 (71.6)	399 (71.6)			166 (5.5)	49 (12.0)	88 (12.1)	46 (24.3)	
Ever drinkers	2,289 (46.3)	456 (38.2)	264 (28.5)	158 (28.4)			2843 (94.5)	359 (88.0)	641 (87.9)	143 (75.7)	
Smoking status, n (%)					< 0.0001						< 0.0001
Never smokers	2,553 (51.6)	710 (59.5)	673 (72.5)	434 (77.8)			1,301 (40.9)	150 (33.0)	339 (40.8)	74 (31.4)	
Previous smokers	481 (9.73)	120 (10.1)	37 (4.0)	23 (4.1)			1,468 (46.2)	204 (44.8)	377 (45.4)	113 (47.9)	
Current smokers	1,912 (38.7)	364 (30.5)	218 (23.5)	101 (18.1)			411 (12.9)	101 (22.2)	114 (13.7)	49 (20.8)	
Hypertension, n (%)					< 0.0001						< 0.0001
No	3957 (80.2)	770 (64.9)	784 (84.8)	391 (70.8)			2240 (70.4)	223 (49.0)	551 (66.3)	98 (41.5)	
Yes	975 (19.8)	417 (35.1)	141 (15.2)	161 (29.2)			941 (29.6)	232 (51.0)	280 (33.7)	138 (58.5)	
BMI (kg/m^2^), mean (SD)	23.2 (3.7)	23.0 (3.9)	22.5 (3.8)	22.6 (4.1)	< 0.0001		27.5 (4.6)	29.8 (5.7)	27.3 (4.5)	29.1 (5.4)	< 0.0001

Abbreviations: CHARLS, China Health and Retirement Longitudinal Study; ELSA, English Longitudinal Study of Ageing; SD, standard deviation

Numbers may not sum to total due to missing data. Continuous variables were reported as means with SD, while categorical variables were presented as frequencies and percentages. Group differences were examined using analysis of variance (ANOVA) or the Chi-square test, as appropriate

### Association of cognitive frailty with CMD outcomes

Table 2 presents the associations of cognitive and frailty status with the risk of three cardiometabolic outcomes in the combined cohorts. Compared with normal participants, those with cognitive frailty had significantly higher risks across all outcomes after full adjustment for covariates. Specifically, cognitive frailty was associated with increased risks of CMDs (HR 1.58; 95% CI 1.39–1.79), CVDs (HR 1.64; 95% CI 1.42–1.89), and diabetes (HR 1.39; 95% CI 1.11–1.75). Frailty alone was also significantly associated with increased risks of CMDs (HR 1.53; 95% CI 1.39–1.67), CVDs (HR 1.48; 95% CI 1.34–1.65), and diabetes (HR 1.55; 95% CI 1.32–1.82). In contrast, cognitive impairment alone was not significantly associated with any of the outcomes. Direct comparisons further showed that individuals with cognitive frailty had a significantly higher risk of CMDs and CVDs than those with cognitive impairment only (CMDs: HR 1.48, 95% CI 1.28–1.72; CVDs: HR 1.62, 95% CI 1.36–1.92), whereas the excess risk for diabetes was not statistically significant (HR 1.21, 95% CI 0.93–1.58). No significant differences were observed between cognitive frailty and frailty alone across any of the three outcomes. The distribution of these three cardiometabolic outcomes by cognitive–frailty group in the combined cohorts is presented in Fig. S2.

**Table 2:** Associations of cognitive and frailty status with incident cardiometabolic outcomes in the combined CHARLS and ELSA cohorts

Group	Cases, *n* (%)	CMDs^a^		CVDs		Diabetes	
		Model 1^b^ HR (95%CI)	Model 2^c^ HR (95%CI)	Model 1 HR (95%CI)	Model 2 HR (95%CI)	Model 1 HR (95%CI)	Model 2 HR (95%CI)
**Individual and combined effect**							
Normal	8,129 (65.9%)	Ref.	Ref.	Ref.	Ref.	Ref.	Ref.
Frailty only	1,649 (13.4%)	**1.87 (171**,** 2.04)**	**1.53 (1.39**,** 1.67)**	**1.81 (1.64**,** 2.00)**	**1.48 (1.34**,** 1.65)**	**1.88 (1.62**,** 2.19)**	**1.55 (1.32**,** 1.82)**
Cognitive impairment only	1,759 (14.3%)	1.03 (0.94, 1.14)	1.06 (0.96, 1.18)	0.97 (0.86, 1.08)	1.01 (0.89, 1.14)	1.15 (0.97, 1.36)	1.15 (0.96, 1.38)
Cognitive frailty	794 (6.4%)	**1.91 (1.70**,** 2.16)**	**1.58 (1.39**,** 1.79)**	**1.94 (1.69**,** 2.22)**	**1.64 (1.42**,** 1.89)**	**1.74 (1.40**,** 2.16)**	**1.39 (1.11**,** 1.75)**
**Combined effect vs. individual effect**							
Cognitive frailty vs. frailty only	NA	1.02 (0.90, 1.17)	1.03 (0.90, 1.19)	1.07 (0.92, 1.25)	1.10 (0.94, 1.29)	0.92 (0.73, 1.17)	0.90 (0.70, 1.15)
Cognitive frailty vs. cognitive impairment only	NA	**1.85 (1.61**,** 2.14)**	**1.48 (1.28**,** 1.72)**	**2.01 (1.70**,** 2.37)**	**1.62 (1.36**,** 1.92)**	**1.51 (1.18**,** 1.95)**	1.21 (0.93, 1.58)

CHARLS, China Health and Retirement Longitudinal Study; ELSA, English Longitudinal Study of Ageing; CMDs, cardiometabolic diseases; CVDs, cardiovascular diseases; HR, hazard ratio; NA, not applicable

^a^ CMDs were defined as the presence of either CVDs or diabetes

^b^ Model 1 adjusted for age and gender

^c^ Model 2 adjusted for age, sex, study region, marital status, education level, smoking status, alcohol consumption, and hypertension

### Cohort-specific analyses

**Fig. 1:**
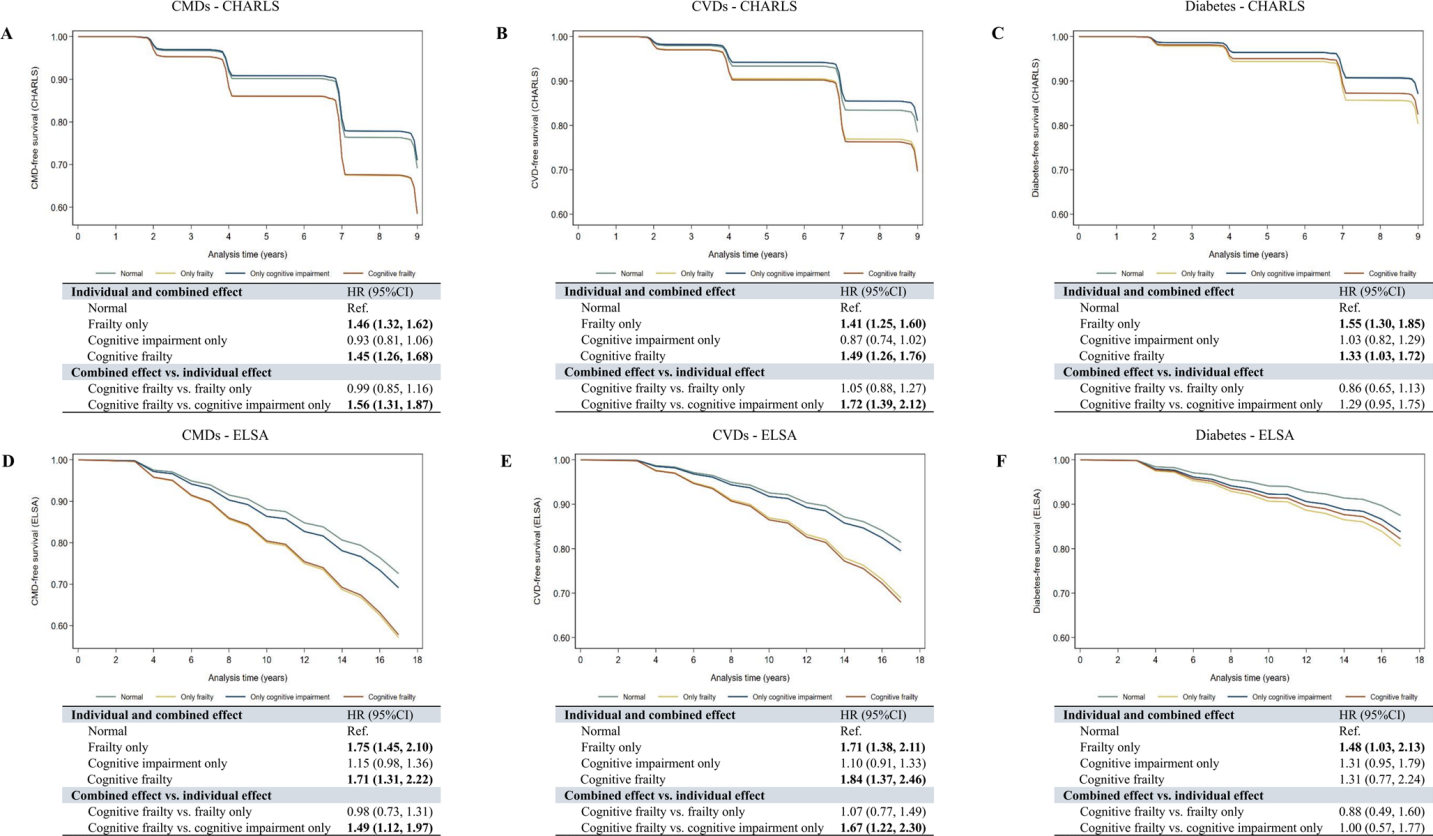
Adjusted survival curves for cardiometabolic outcomes by cognitive and frailty status in the CHARLS and ELSA cohorts. Adjusted survival curves for incident cardiometabolic diseases (CMDs), cardiovascular diseases (CVDs), and diabetes by cognitive and frailty status. Panels **A–C** present results from the China Health and Retirement Longitudinal Study (CHARLS); panels **D–F** present results from the English Longitudinal Study of Ageing (ELSA). Models were adjusted for age, sex, marital status, education level, smoking status, alcohol consumption, and hypertension. HR, hazard ratio; CI, confidence interval

Figure 1 presents adjusted survival curves and corresponding HRs for CMDs, CVDs, and diabetes by cognitive–frailty groups in CHARLS (panels A–C) and ELSA (panels D–F). In both cohorts, compared with the normal group, cognitive frailty was significantly associated with higher risks of CMDs and CVDs. In CHARLS, cognitive frailty was also linked to increased diabetes risk (HR 1.33, 95% CI 1.03–1.72), whereas the association was not significant in ELSA (HR 1.31, 95% CI 0.77–2.24). Frailty alone was consistently associated with higher risks of all three outcomes across both cohorts, while cognitive impairment alone was not significantly associated with any outcome. Compared to cognitive impairment alone, cognitive frailty conferred significantly greater risks of CMDs and CVDs in both cohorts, but not for diabetes.

### Subgroup analyses by sex and age

**Fig. 2:**
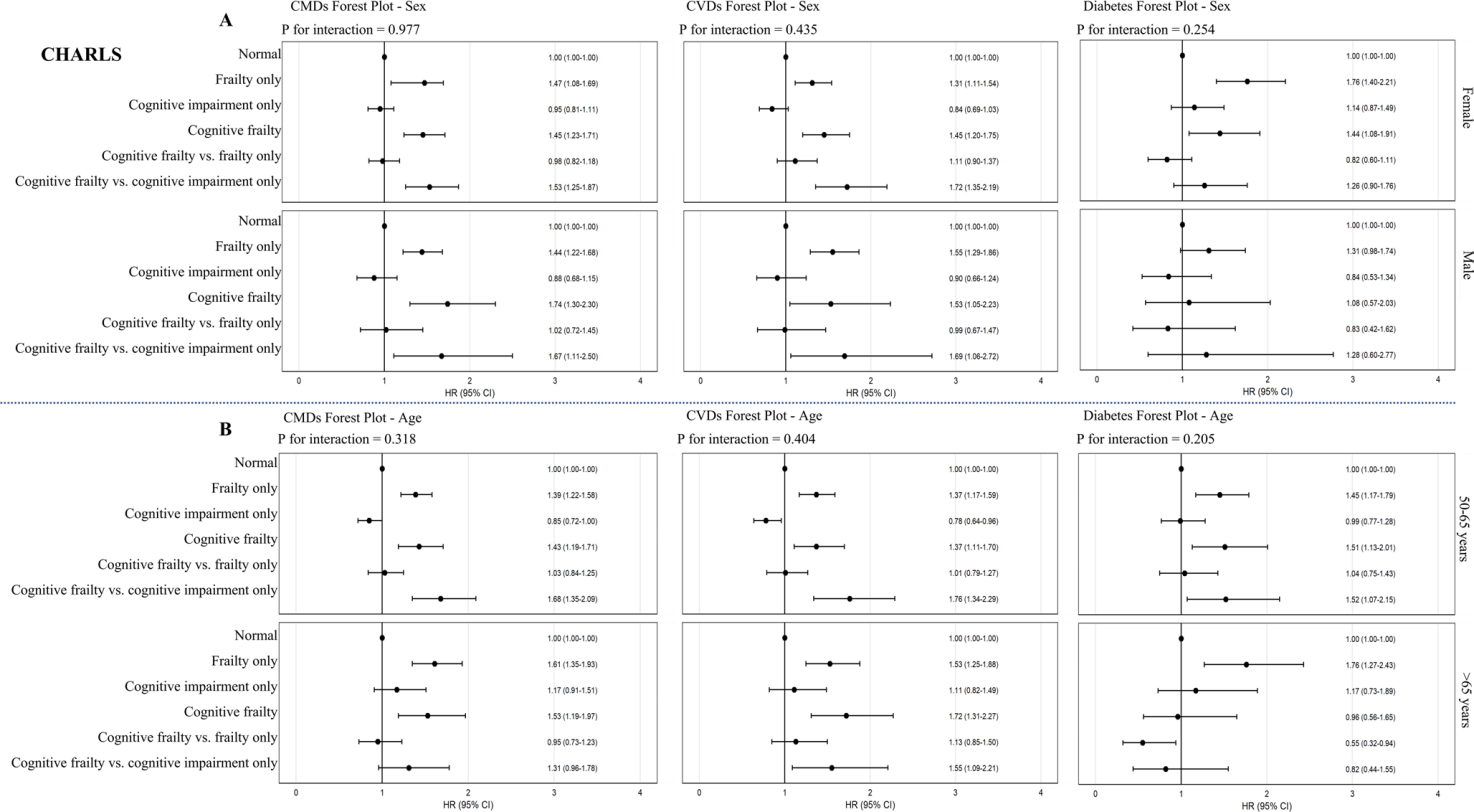
Subgroup analyses of the associations between cognitive and frailty status and cardiometabolic outcomes by sex and age in CHARLS. Forest plots showing hazard ratios (HRs) and 95% confidence intervals (CIs) for incident cardiometabolic diseases (CMDs), cardiovascular diseases (CVDs), and diabetes across cognitive and frailty status categories, stratified by sex (Panel **A**) and age group (Panel **B**) among the China Health and Retirement Longitudinal Study (CHARLS) participants. Interaction *P* values indicate effect modification by subgroup. Models adjusted for age (in sex-stratified models), sex (in age-stratified models), marital status, education level, smoking status, alcohol consumption, and hypertension

**Fig. 3:**
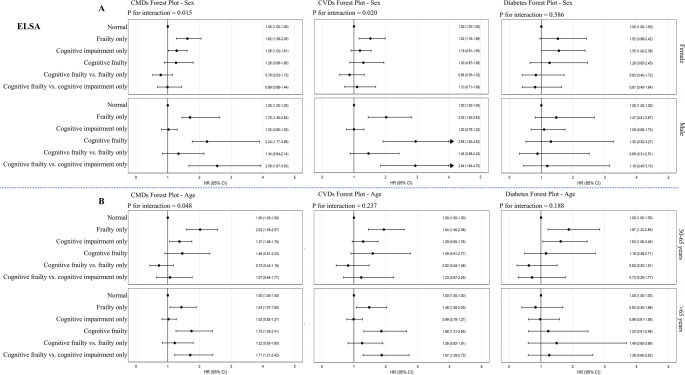
Subgroup analyses of the associations between cognitive and frailty status and cardiometabolic outcomes by sex and age in ELSA. Forest plots showing hazard ratios (HRs) and 95% confidence intervals (CIs) for incident cardiometabolic diseases (CMDs), cardiovascular diseases (CVDs), and diabetes across cognitive and frailty status categories, stratified by sex (Panel **A**) and age group (Panel **B**) among English Longitudinal Study of Ageing (ELSA) participants. Interaction *P* values indicate effect modification by subgroup. Models adjusted for age (in sex-stratified models), sex (in age-stratified models), marital status, education level, smoking status, alcohol consumption, and hypertension

Subgroup analyses by sex and age group are presented in Fig. 2 (CHARLS) and Fig. 3 (ELSA). In CHARLS, no significant interactions were found between sex or age group and cognitive–frailty status for any of the outcomes (all *P* for interaction > 0.05). In contrast, significant effect modification by sex and age was observed in ELSA. Specifically, the association between cognitive frailty and CMDs or CVDs was significantly stronger in males than in females (*P* for interaction = 0.015 for CMDs, 0.020 for CVDs). Similarly, the association between cognitive frailty and CMDs varied significantly by age group (*P* for interaction = 0.048), with higher hazard ratios observed in participants aged > 65 years. No significant interactions were identified for diabetes in either cohort.

### Restricted cubic spline analyses 

**Fig. 4:**
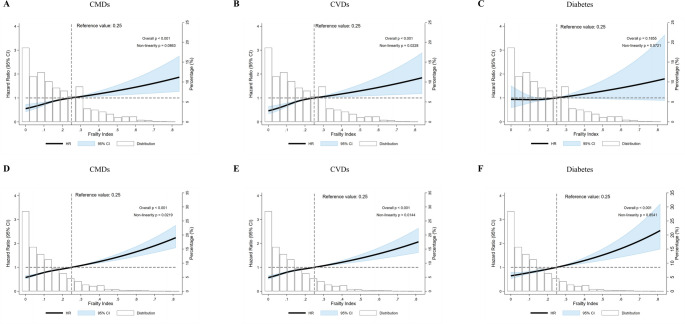
Dose–response associations between FI and cardiometabolic outcomes among cognitively impaired individuals in the combined CHARLS and ELSA cohorts. Restricted cubic spline models showing the association between frailty index (FI) and risk of cardiometabolic diseases (CMDs, panels **A** and **D**), cardiovascular diseases (CVDs, panels **B** and **E**), and diabetes (panels **C** and **F**) among participants with (panels **A–C**) and without (panels **D–F**) cognitive impairment from the combined cohort. Models were adjusted for age, sex, study region, marital status, education level, smoking status, alcohol consumption, and hypertension. The reference value was set at FI = 0.25. Shaded areas represent 95% confidence intervals. Bars indicate the distribution of FI. *P* values indicate overall association and test for non-linearity

Among participants with cognitive impairment (Fig. 4, panels A–C), a significant positive association was observed between FI and the risks of CMDs and CVDs (overall *P* < 0.001 for both), with evidence of non-linearity for CVDs (*P* for non-linearity = 0.0228). The association with diabetes followed a similar trend but did not reach statistical significance (overall *P* = 0.1855). Among participants without cognitive impairment (Fig. 4, panels D–F), comparable dose–response relationships were observed for CMDs and CVDs (overall *P* < 0.001 for both), with significant non-linearity in both associations (*P* for non-linearity < 0.05). Notably, the association between FI and diabetes was statistically significant in this subgroup (overall *P* < 0.001), although no evidence of non-linearity was found (*P* = 0.8541).

### Sensitivity analyses

Results from Fine–Gray models were consistent with the main analyses, with cognitive frailty remaining significantly associated with increased risks of CMDs, CVDs, and diabetes (Tables S4–S6). When frailty was defined using the PFP, cognitive frailty remained significantly associated with increased risks of CMDs and CVDs, but not diabetes, after full adjustment for covariates (Table S7). Notably, in contrast to the main analysis using the FI, cognitive impairment alone was modestly but significantly associated with higher risks of CMDs (HR 1.10, 95% CI 1.01–1.20). No significant association was observed between cognitive impairment alone and diabetes or CVDs. The overall pattern of results remained broadly consistent with the main results. Cognitive frailty was associated with higher risks of CMDs and CVDs than cognitive impairment alone, although the differences did not reach statistical significance. As shown in Tables S8 and S9, the associations between cognitive frailty and CMD outcomes remained directionally consistent in both cohorts, despite variations in statistical significance.

## Discussion

In this large, population-based study involving cohorts from China and the UK, we found that individuals with cognitive frailty had significantly higher risks of CMDs, CVDs, and diabetes compared with those with normal cognitive function and frailty status, based on our primary analyses using the FI. These associations were consistently observed across both cohorts and remained robust after adjustment for demographic, behavioral, and clinical covariates. Notably, frailty alone was also associated with elevated risks of all three outcomes, while cognitive impairment alone was not significantly associated with any. Subgroup analyses further revealed effect modification by sex and age in ELSA, but not in CHARLS. Furthermore, results remained comparable in sensitivity analyses using competing risk models and alternative frailty definitions.

One notable finding was that cognitive impairment, in the absence of frailty, may not substantially elevate cardiometabolic risk. Individuals who retain physical robustness may be better able to engage in self-care and maintain healthy behaviors, thereby mitigating the adverse impact of cognitive decline. This behavioral compensation may be further supported by the concept of cognitive reserve, which posits that individuals with greater reserve can better tolerate neuropathological changes while maintaining functional independence [28]. Even in the presence of measurable cognitive deficits, preserved cognitive reserve and physical function may allow older adults to effectively self-manage chronic conditions through medications adherence, dietary regulation, and healthcare service engagement. An alternative explanation is that some cases of cognitive impairment may have been transient or subclinical, thereby attenuating their long-term associations with cardiometabolic outcomes [29]. Interestingly, the addition of cognitive impairment to frailty did not significantly increase risk beyond that conferred by frailty alone. This may suggest that frailty alone captures much of the systemic vulnerability relevant to cardiometabolic risk, thereby limiting the incremental contribution of cognitive deficits. Subgroup analyses revealed effect modification by sex and age in ELSA but not in CHARLS. In ELSA, the association between cognitive frailty and CMDs was stronger among men and individuals aged > 65 years. This is consistent with previous research reporting stronger associations between cognitive frailty and mortality among older adults (≥ 70 years) and males [16]. It is plausible that older individuals with cognitive frailty are more susceptible to adverse cardiometabolic outcomes due to accumulated physiological burden, multisystem dysregulation, and reduced resilience with age, whereas younger individuals may still retain greater functional reserves that mitigate such risks. While the reasons for the sex-specific differences remain unclear, these findings underscore the need for further research into how biological, behavioral, and social factors may differentially influence cardiometabolic risk across population subgroups. The absence of interaction effects in CHARLS may reflect more uniform risk distributions or population-specific differences in the expression of frailty. Furthermore, RCS analysis demonstrated significant dose–response associations between the FI and the risks of CMDs and CVDs across both cognitive subgroups. While similar but non-significant trends were observed for diabetes among individuals with cognitive impairment, the association reached statistical significance among those without cognitive impairment. These findings reinforce the role of frailty as a fundamental determinant of cardiometabolic risk, regardless of cognitive status. Notably, the risk elevation was evident even at FI values below the conventional frailty threshold (FI = 0.25), suggesting that individuals not yet classified as frail may still be at increased cardiometabolic risk. This highlights the potential utility of FI for identifying at-risk individuals earlier along the frailty continuum. Additionally, the sensitivity analysis using the PFP showed a significant association between cognitive impairment and CMDs, which was not observed in the FI-based analysis. This discrepancy may reflect differences in the domains captured by each construct. PFP focuses on specific physical function components—such as slowness, weakness, and exhaustion—that may be more directly related to cardiometabolic dysregulation [20]. In contrast, FI includes a broader range of deficits [19], some of which may be less strongly linked to CMDs pathophysiology.

The mechanisms underlying the observed associations between cognitive frailty and cardiometabolic risk are likely to be multifactorial. Cognitive frailty reflects the co-occurrence of neurocognitive decline and physical vulnerability, both of which have been linked to pathophysiological mechanisms such as chronic systemic inflammation, mitochondrial dysfunction, and oxidative stress [7, 30]. These mechanisms may accelerate vascular aging, impair glucose metabolism, and promote atherosclerosis, thereby increasing susceptibility to both CVDs and diabetes [31–34]. Furthermore, individuals with cognitive frailty may be less likely to adhere to medical treatment, maintain physical activity, or follow dietary recommendations [2], thereby further exacerbating cardiometabolic risk.

This study has several strengths. First, we utilized data from two nationally representative cohorts, enhancing generalizability and cross-cultural relevance. Second, the prospective design, large sample size, and long follow-up enabled robust risk estimation. Third, comprehensive adjustment for covariates and multiple sensitivity analyses—including competing risk models and alternative frailty definitions—supported the robustness of our findings. Nonetheless, several limitations warrant consideration. First, cognitive impairment and frailty were assessed at a single time point, limiting insight into their temporal progression. Second, residual confounding from unmeasured factors, such as dietary patterns, may persist. Third, minor differences in variable operationalization across cohorts could have introduced heterogeneity; for example, slight variations existed in the definition of inactivity in the PFP between cohorts. However, the overall approach remained harmonized to allow meaningful cross-cohort comparison. Fourth, cardiometabolic outcomes were based on self-reported physician diagnoses. The accuracy of such reporting may vary across subpopulations; for example, individuals from rural areas in China or socioeconomically disadvantaged groups in the UK may underreport diagnoses due to limited healthcare access. This differential misclassification could lead to attenuated associations. Future studies incorporating objective biomarkers such as glycated hemoglobin (HbA1c) or fasting glucose for diabetes are warranted to validate self-reported cardiometabolic outcomes and reduce potential misclassification bias. Fifth, in ELSA, mortality data were unavailable beyond wave 6, which may have affected follow-up estimates and the accuracy of competing risk adjustment [21]. Sixth, a proportion of participants were lost to follow-up in both cohorts. Although we described the baseline differences between groups to support interpretation of findings, the precise impact of attrition on study estimates could not be definitively assessed. Additionally, participants with insufficient data for frailty assessment were excluded to ensure the validity of frailty classification. While this approach minimizes measurement error, it may introduce selection bias if excluded individuals systematically differed from those included. Finally, as with all observational studies, causal inference cannot be established.

## Conclusions

Cognitive frailty was independently associated with an elevated risk of CMDs, particularly CVDs, in both Chinese and UK cohorts. These associations were stronger than those observed for cognitive impairment alone and remained robust across alternative frailty definitions and analytical approaches. The inclusion of culturally diverse populations enhances the generalizability of our findings and underscores the need for integrated, contextually appropriate strategies to identify and address both cognitive impairment and frailty in aging populations.

## Supplementary Information

Below is the link to the electronic supplementary material.

Supplementary Material 1

## Data Availability

The CHARLS data are available upon request (https://charls.pku.edu.cn/), while the ELSA data are available after registration (https://beta.ukdataservice.ac.uk/datacatalogue/series/series?id=200011).
