# Supplementary material for: Cognitive frailty and cardiometabolic risk in middle-aged and older adults: evidence from the UK and China
Source: Aging Clin Exp Res. 2025 Sep 4;37(1):269. doi: 10.1007/s40520-025-03179-1 (PMC12411595; doi:10.1007/s40520-025-03179-1)
Supplement: Supplementary file 1 — Supplementary Material 1 [file 40520_2025_3179_MOESM1_ESM.docx]

Cognitive Frailty and Cardiometabolic Risk in Middle-Aged and Older Adults:

Evidence from the UK and China

Haiyang Yan ^1^, Jingjing Lang ^2^, Chengfeng Li ^3^, Samaneh Eftekhariranjbar ^4,5^, Guoyan Jiang ^1^, Jing Lei ^6^, Lixin Sun ^7^, Carlos J. Toro-Huamanchumo ^3,8,9^, Zhongyang Guan ^10,11^

^1^ Yancheng Municipal Center for Disease Control and Prevention, Yancheng, Jiangsu, China

^2^ Children’s Hospital of Soochow University, Suzhou, Jiangsu, China

^3^ Nutrition and Health Innovation Research Institute, School of Medical and Health Sciences, Edith Cowan University, Western Australia, Australia.

^4^ University G. D'Annunzio of Chieti-Pescara, Chieti, Italy

^5^ Sapienza University of Rome, Rome, Italy

^6^ Curtin Medical School, Faculty of Health Science, Curtin University, Perth, Western Australia, Australia

^7^ Center for Health Policy and Development Research, Jiangxi Science and Technology Normal University, Nanchang, Jiangxi, China

^8^ OBEMET Center for Obesity and Metabolic Health, Lima, Peru

^9^ Research Unit for Health Evidence Generation and Synthesis, Universidad San Ignacio de Loyola, Lima, Peru

^10^ School of Population Health, Faculty of Health Sciences, Curtin University, Perth, Western Australia, Australia

^11^ Dementia Centre of Excellence, enAble Institute, Curtin University, Perth, Western Australia, Australia

**Address correspondence to:**

Zhongyang Guan, School of Population Health, Faculty of Health Science, Curtin University, Perth, WA, Australia. Email: [zhongyang.guan@postgrad.curtin.edu.au](mailto:zhongyang.guan@postgrad.curtin.edu.au)

ORCID：[0000-0003-0737-3929](https://orcid.org/0000-0003-0737-3929)

Haiyang Yan and Jingjing Lang contributed equally to this work and shared first authorship.

**Target journal:** Aging Clinical and Experimental Research

**CONTENTS**

**Supplementary Methods** 3

**Fig. S1.** Selection process for the study population 4

**Fig. S2.** Distribution of outcome events in two cohorts combined 5

**Table S1.** The items used to construct FI 6

**Table S2.** Baseline characteristics of all participants with available baseline data by follow-up status 7

**Table S3.** Baseline characteristics of participants excluded due to insufficient frailty data 8

**Table S4.** Association of frailty (identified by FI) and cognitive impairment with CMD outcomes in the combined CHARLS and ELSA cohorts by Fine-Gray model 9

**Table S5.** Association of frailty (identified by FI) and cognitive impairment with CMD outcomes by Fine-Gray model (CHARLS) 10

**Table S6.** Association of frailty (identified by FI) and cognitive impairment with CMD outcomes by Fine-Gray model (ELSA) 11

**Table S7.** Association of frailty (identified by PFP) and cognitive impairment with CMD outcomes in the combined CHARLS and ELSA cohorts 12

**Table S8.** Association of frailty (identified by PFP) and cognitive impairment with CMD outcomes (CHARLS) 13

**Table S9.** Association of frailty (identified by PFP) and cognitive impairment with CMD outcomes

(ELSA) 14

**Supplementary Methods**

Study design of CHARLS and ELSA

CHARLS, initiated in 2011, employed a multistage, stratified, probability-proportional-to-size sampling method to recruit community-dwelling adults aged 45 years or older from 28 provinces across China (N = 17,708) [1]. Participants were followed up in 2013, 2015, 2018, and 2020, comprising a total of five waves (2011–2020). ELSA, which commenced in 2002–2003, recruited community-dwelling adults aged 50 years and older in England who had previously participated in the 1998, 1999, or 2001 Health Survey for England (N = 12,099) [2]. Participants were followed up biennially through 2020, with 10 waves of data collected. In the current study, baseline data were collected from wave 1 of CHARLS (2011) and wave 2 of ELSA (2004–2005), which was the first ELSA wave that included all baseline variables necessary for this analysis. Follow-up surveys continued through subsequent waves until wave 5 of CHARLS (2020) and wave 9 of ELSA (2018–2019).

PFP Assessment

The adapted PFP approach includes five criteria: shrinking, weakness, exhaustion, slowness, and inactivity [3, 4]. Shrinking was defined as a loss of 5% of body weight in the previous year (for CHARLS participants only) or a BMI of ≤ 18.5 kg/m² (for both CHARLS and ELSA participants). Weakness was defined as the lowest quintile of maximum grip strength (either hand), stratified by sex within each BMI quartile. Exhaustion was defined by a positive response to either of the two questions: “Felt that everything I did was an effort in the last week” or “Could not get going in the last week”, from the Center for Epidemiologic Studies—Depression Scale (CES-D). Slowness was identified as the lowest quintile of the mean walking speed across two normal-paced walk tests, adjusted for sex and standing height. For CHARLS participants, inactivity was defined as not engaging in any physical activity of any intensity (vigorous, moderate, or mild) for at least 10 continuous minutes in a typical week. For ELSA participants, physical inactivity was defined as never or hardly ever engaging in physical activity at any intensity. Given the limited data availability in CHARLS, participants who met three or more criteria were defined as frail; otherwise, they were considered non-frail. We excluded participants with missing data for four of the five PFP criteria [5].

**Reference**

1. Zhao Y, Hu Y, Smith JP, Strauss J, Yang G (2014) Cohort profile: the China health and retirement longitudinal study (CHARLS). Int J Epidemiol 43(1):61-8. <https://doi.org/10.1093/ije/dys203>
2. Steptoe A, Breeze E, Banks J, Nazroo J (2013) Cohort profile: the English longitudinal study of ageing. Int J Epidemiol 42(6):1640-8. <https://doi.org/10.1093/ije/dys168>
3. Fried LP, Tangen CM, Walston J, Newman AB, Hirsch C, Gottdiener J, et al (2001) Frailty in older adults: evidence for a phenotype. The journals of gerontology series a: biological sciences and medical sciences 56(3):M146-M57. <https://doi.org/10.1093/gerona/56.3.M146>
4. Leme DEdC, De Oliveira C (2023) Machine learning models to predict future frailty in community-dwelling middle-aged and older adults: the ELSA cohort study. The Journals of Gerontology: Series A 78(11):2176-84. <https://doi.org/10.1093/gerona/glad127>
5. Chen C, Park J, Wu C, Xue Q, Agogo G, Han L, et al. (2020) Cognitive frailty in relation to adverse health outcomes independent of multimorbidity: results from the China health and retirement longitudinal study. Aging 12(22):23129. [https://doi.org/10.18632/aging.104078](%20https:/doi.org/10.18632/aging.104078)

**Fig. S1.** Selection process for the study population.


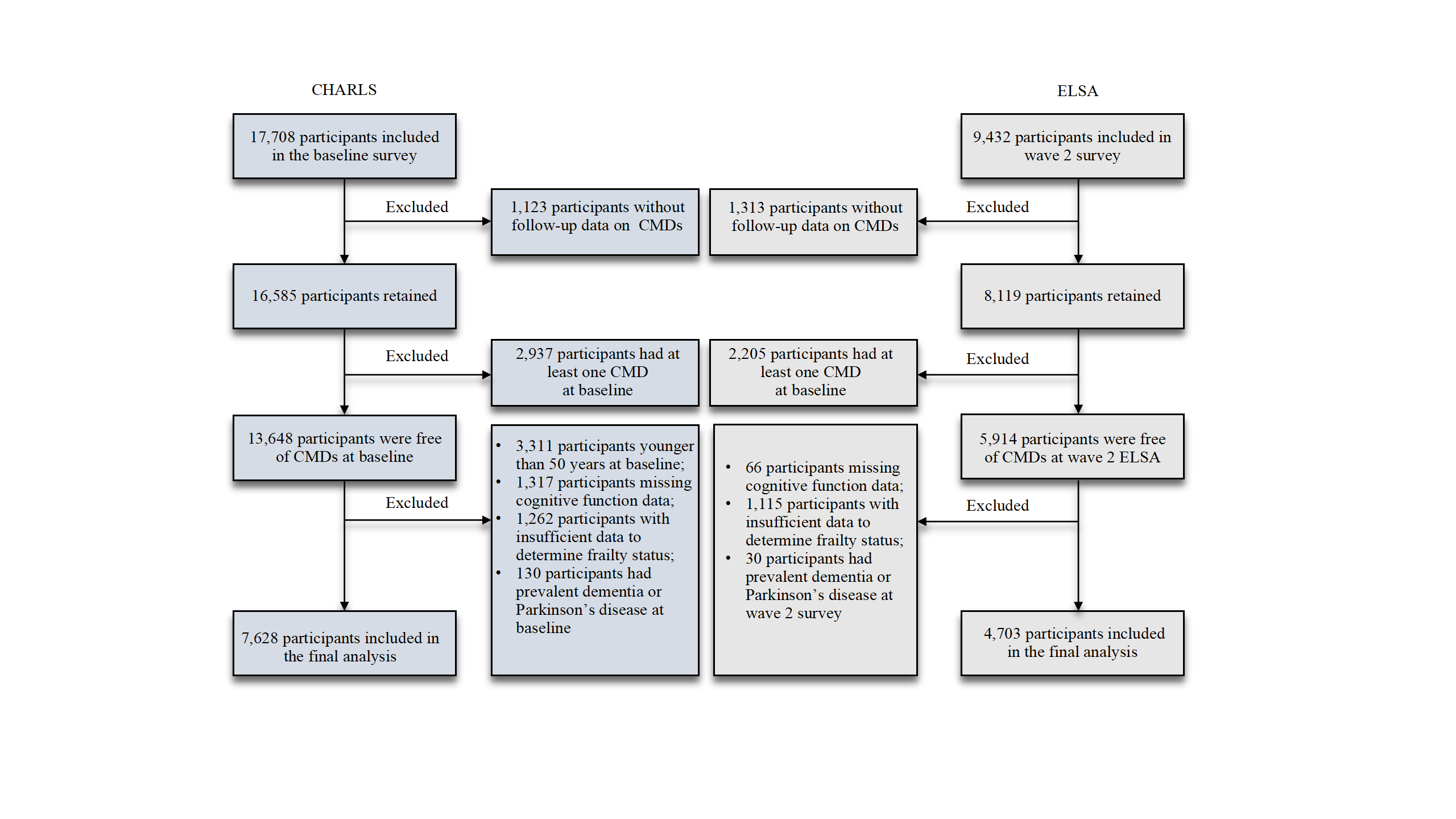


CHARLS, China Health and Retirement Longitudinal Study; ELSA, English Longitudinal Study of Ageing; CMDs, cardiometabolic diseases.


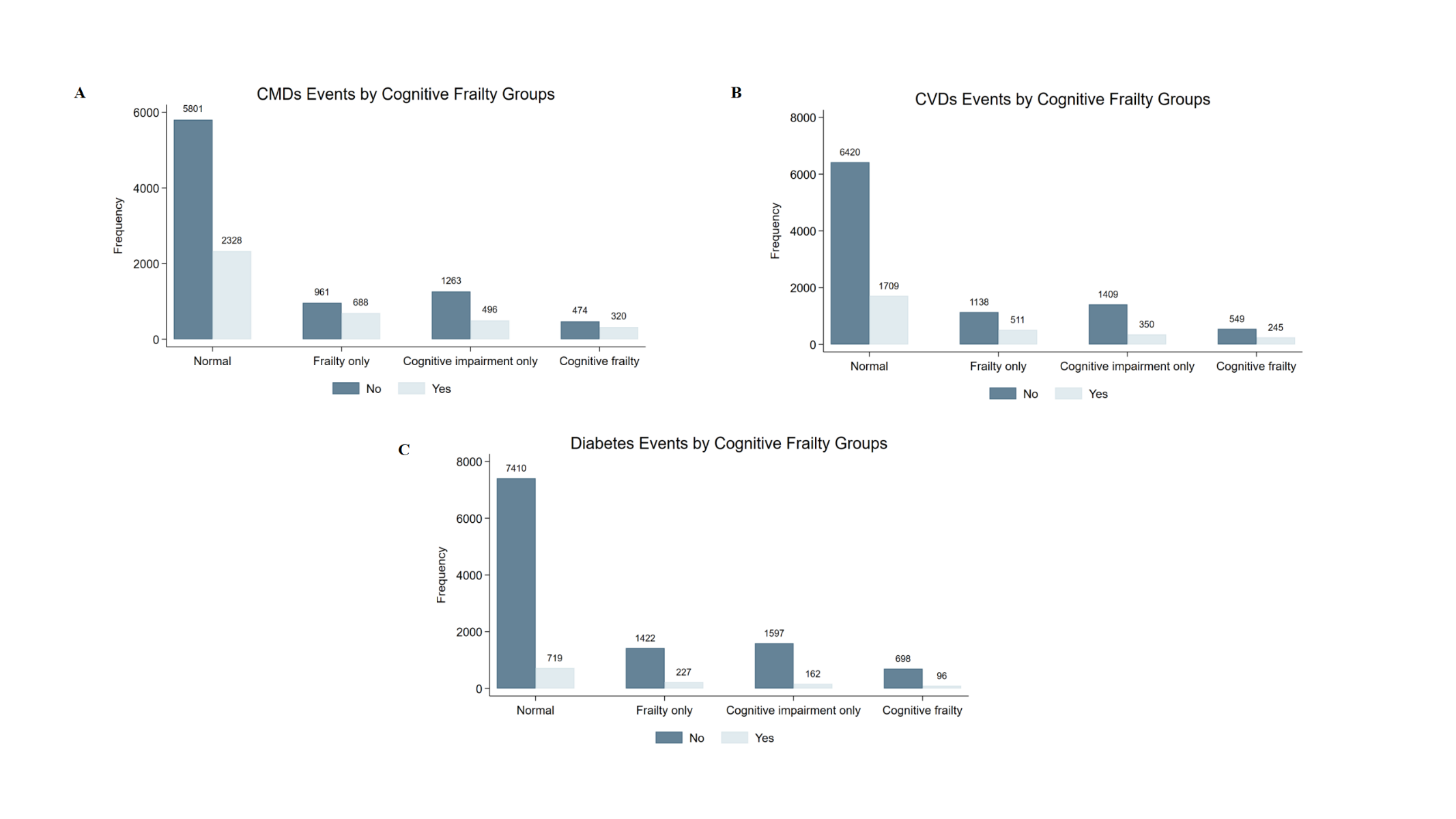
**Fig. S2.** Distribution of outcome events in two cohorts combined.

CMDs, cardiometabolic diseases; CVDs, cardiovascular diseases.

**Table S1.** The items used to construct the frailty index Frailty index

| Description of the items | | Cut-off value |
| --- | --- | --- |
| 1 | Self-reported general health status | Very poor or poor = 1,  Very good, good, or fair = 0 |
| 2 | Self reported physician diagnosed cancer or malignant tumour | Yes = 1, No = 0 |
| 3 | Self reported physician diagnosed chronic lung disease | Yes = 1, No = 0 |
| 4 | Self reported physician diagnosed emotional, nervous, psychiatric problems | Yes = 1, No = 0 |
| 5 | Self reported physician diagnosed arthritis | Yes = 1, No = 0 |
| 6 | Difficulty with dressing | Yes = 1, No = 0 |
| 7 | Difficulty with bathing or showering | Yes = 1, No = 0 |
| 8 | Difficulty with eating | Yes = 1, No = 0 |
| 9 | Difficulty with getting in and out of bed | Yes = 1, No = 0 |
| 10 | Difficulty with using the toilet | Yes = 1, No = 0 |
| 11 | Difficulty with managing money | Yes = 1, No = 0 |
| 12 | Difficulty with taking medication | Yes = 1, No = 0 |
| 13 | Difficulty with shopping for groceries | Yes = 1, No = 0 |
| 14 | Difficulty with preparing meals | Yes = 1, No = 0 |
| 15 | Difficulty with walking 100 yards or one block | Yes = 1, No = 0 |
| 16 | Difficulty with getting up from a chair after sitting for long periods | Yes = 1, No = 0 |
| 17 | Difficulty with climbing several flights of stairs without resting | Yes = 1, No = 0 |
| 18 | Difficulty with stooping, kneeling, or crouching | Yes = 1, No = 0 |
| 19 | Difficulty with lifting or carrying weights over 10 pounds/jins | Yes = 1, No = 0 |
| 20 | Difficulty with picking up a coin from the table | Yes = 1, No = 0 |
| 21 | Difficulty with reaching arms above shoulder level | Yes = 1, No = 0 |
| 22 | Feeling depressed most of time | Yes = 1, No = 0 |
| 23 | Feeling that everything is an effort most of time | Yes = 1, No = 0 |
| 24 | Feeling that sleep was restless most of time | Yes = 1, No = 0 |
| 25 | Feeling happy most of time | No = 1, Yes = 0 |
| 26 | Feeling enjoying life most of time | No = 1, Yes = 0 |

**Table S2.** Baseline characteristics of all participants with available baseline data by follow-up status

|  | **CHARLS (n=17,708)** | | |  | **ELSA (n=9,432)** | | |
| --- | --- | --- | --- | --- | --- | --- | --- |
|  | Completed follow-up  (n = 16,585; 93.7%) | Lost to follow-up  (n = 1,123; 6.3%) | *P* value |  | Completed follow-up  (n = 8,119; 86.1%) | Lost to follow-up  (n = 1,313; 13.9%) | *P* value |
| Age (years), mean (SD) | 58.6 (9.9) | 63.8 (12.6) | <0.0001 |  | 65.2 (10.2) | 68.5 (11.8) | <0.0001 |
| Sex, n (%) |  |  | 0.002 |  |  |  | 0.086 |
| Female | 8,693 (52.4) | 534 (47.6) |  |  | 4,596 (56.6) | 710 (54.1) |  |
| Male | 7,892 (47.6) | 587 (52.4) |  |  | 3,523 (43.4) | 603 (45.9) |  |
| Education, n (%) |  |  | <0.0001 |  |  |  | <0.0001 |
| High school not completed | 14,614 (88.1) | 911 (82.2) |  |  | 3,332 (44.9) | 730 (60.9) |  |
| High school or above | 1,971 (11.9) | 197 (17.8) |  |  | 4,097 (55.2) | 469 (39.1) |  |
| Marital status, n (%) |  |  | <0.0001 |  |  |  | 0.003 |
| Married or partnered | 14,643 (88.4) | 857 (77.1) |  |  | 5,443 (67.1) | 826 (62.9) |  |
| Others | 1,921 (11.6) | 254 (22.9) |  |  | 2,675 (33.0) | 487 (37.1) |  |
| Alcohol consumption, n (%) |  |  | 0.428 |  |  |  | 0.002 |
| Never drinkers | 10,071 (61.2) | 647 (60.0) |  |  | 750 (10.5) | 133 (13.8) |  |
| Ever drinkers | 6,391 (38.8) | 432 (40.0) |  |  | 6,427 (89.6) | 834 (86.3) |  |
| Smoking status, n (%) |  |  | <0.0001 |  |  |  | <0.0001 |
| Never smokers | 10,586 (64.2) | 685 (63.4) |  |  | 3,062 (37.7) | 416 (31.7) |  |
| Previous smokers | 1,297 (7.9) | 120 (11.1) |  |  | 3,831 (47.2) | 651 (49.6) |  |
| Current smokers | 4,596 (27.9) | 275 (25.5) |  |  | 1,225 (15.1) | 246 (18.7) |  |
| Hypertension, n (%) |  |  | <0.0001 |  |  |  | <0.0001 |
| No | 12,219 (74.5) | 716 (66.4) |  |  | 4,878 (60.1) | 704 (53.6) |  |
| Yes | 4,173 (25.5) | 362 (33.6) |  |  | 3,240 (39.9) | 609 (46.4) |  |
| BMI (kg/m^2^), mean (SD) | 23.5 (3.8) | 22.7 (3.9) | <0.0001 |  | 27.9 (4.9) | 27.8 (5.0) | 0.6366 |

Abbreviations: CHARLS, China Health and Retirement Longitudinal Study; ELSA, English Longitudinal Study of Ageing; SD, standard deviation.

Numbers may not sum to total due to missing data. Continuous variables were reported as means with SD, while categorical variables were presented as frequencies and percentages. Group differences were examined using analysis of variance (ANOVA), the Wilcoxon rank-sum test, or the Chi-square test, as appropriate.

**Table S3.** Baseline characteristics of participants excluded due to insufficient frailty data.

|  | CHARLS |  | ELSA |
| --- | --- | --- | --- |
|  | Excluded due to insufficient frailty data (n = 1,262) |  | Excluded due to insufficient frailty data (n = 1,115) |
| Age (years), mean (SD) | 63.2 (12.4) |  | 66.2 (11.5) |
| Sex, n (%) |  |  |  |
| Female | 734 (58.2) |  | 587 (52.7) |
| Male | 528 (41.8) |  | 528 (47.4) |
| Education, n (%) |  |  |  |
| High school not completed | 1158 (92.5) |  | 486 (47.4) |
| High school or above | 94 (7.5) |  | 540 (52.6) |
| Marital status, n (%) |  |  |  |
| Married or partnered | 829 (67.3) |  | 750 (67.3) |
| Others | 402 (32.6) |  | 365 (32.7) |
| Alcohol consumption, n (%) |  |  |  |
| Never drinkers | 731 (66.2) |  | 116 (12.7) |
| Ever drinkers | 374 (33.9) |  | 801 (87.4) |
| Smoking status, n (%) |  |  |  |
| Never smokers | 1003 (79.5) |  | 372 (33.4) |
| Previous smokers | 94 (7.5) |  | 576 (50.9) |
| Current smokers | 165 (13.1) |  | 176 (15.8) |
| Hypertension, n (%) |  |  |  |
| No | 636 (57.6) |  | 580 (52.0) |
| Yes | 467 (42.4) |  | 535 (48.0) |
| BMI (kg/m^2^), mean (SD) | 23.5 (4.2) |  | 28.2 (4.9) |

Abbreviations: CHARLS, China Health and Retirement Longitudinal Study; ELSA, English Longitudinal Study of Ageing; SD, standard deviation.

Numbers may not sum to total due to missing data. Continuous variables were reported as means with SD, while categorical variables were presented as frequencies and percentages.

**Table S4.** Association of physical frailty (identified by FI) and cognitive impairment with CMD outcomes in the combined CHARLS and ELSA cohorts by Fine-Gray model.

| **Group** | **Cases, n (%)** | **CMDs**^a^ | | **CVDs** | | **Diabetes** | |
| --- | --- | --- | --- | --- | --- | --- | --- |
|  |  | Model 1^b^  SHR (95%CI) | Model 2^c^  SHR (95%CI) | Model 1  SHR (95%CI) | Model 2  SHR (95%CI) | Model 1  SHR (95%CI) | Model 2  SHR (95%CI) |
| **Individual and combined effect** |  |  |  |  |  |  |  |
| Normal | 8,129 (65.9%) | Ref. | Ref. | Ref. | Ref. | Ref. | Ref. |
| Frailty only | 1,649 (13.4%) | **1.63 (1.49, 1.77)** | **1.37 (1.24, 1.50)** | **1.58 (1.42, 1.75)** | **1.34 (1.20, 1.49)** | **1.69 (1.45, 1.98)** | **1.41 (1.20, 1.67)** |
| Cognitive impairment only | 1,759 (14.3%) | 1.00 (0.90, 1.10) | 1.01 (0.91, 1.12) | 0.93 (0.83, 1.05) | 0.96 (0.84, 1.08) | 1.14 (0.95, 1.35) | 1.12 (0.93, 1.34) |
| Cognitive frailty | 794 (6.4%) | **1.53 (1.35, 1.73)** | **1.27 (1.11, 1.46)** | **1.53 (1.32, 1.77)** | **1.30 (1.11, 1.53)** | **1.58 (1.27, 1.96)** | **1.27 (1.00, 1.60)** |
| **Combined effect vs. individual effect** |  |  |  |  |  |  |  |
| Cognitive frailty vs. frailty only | NA | 0.94 (0.82, 1.08) | 0.93 (0.80, 1.08) | 0.97 (0.82, 1.14) | 0.97 (0.82, 1.16) | 0.93 (0.73, 1.19) | 0.90 (0.70, 1.16) |
| Cognitive frailty vs. cognitive impairment only | NA | **1.53 (1.32, 1.77)** | **1.26 (1.08, 1.48)** | **1.64 (1.38, 1.95)** | **1.36 (1.13, 1.64)** | **1.39 (1.08, 1.80)** | 1.14 (0.87, 1.49) |

CHARLS, China Health and Retirement Longitudinal Study; ELSA, English Longitudinal Study of Ageing; CMDs, cardio-metabolic diseases; CVDs, cardiovascular diseases; SHR, subdistribution hazard ratio; NA, not applicable.

^a^ CMDs were defined as the presence of either CVDs or diabetes.

^b^ Model 1 adjusted for age and gender.

^c^ Model 2 adjusted for age, sex, study region, marital status, education level, smoking status, alcohol consumption, and hypertension.

**Table S5.** Association of physical frailty (identified by FI) and cognitive impairment with CMD outcomes by Fine-Gray model (CHARLS).

| **Group** | **CMDs**^a^ | **CVDs** | **Diabetes** |
| --- | --- | --- | --- |
|  | SHR (95%CI)^b^ | SHR (95%CI) | SHR (95%CI) |
| **Individual and combined effect** |  |  |  |
| Normal | Ref. | Ref. | Ref. |
| Frailty only | **1.34 (1.20, 1.49)** | **1.30 (1.15, 1.48)** | **1.42 (1.18, 1.70)** |
| Cognitive impairment only | **0.87 (0.76, 1.00)** | **0.81 (0.68, 0.96)** | 0.99 (0.79, 1.25) |
| Cognitive frailty | **1.19 (1.02, 1.38)** | 1.19 (1.00, 1.43) | 1.22 (0.94, 1.58) |
| **Combined effect vs. individual effect** |  |  |  |
| Cognitive frailty vs. frailty only | 0.89 (0.75, 1.05) | 0.92 (0.75, 1.12) | 0.86 (0.65, 1.14) |
| Cognitive frailty vs. cognitive impairment only | **1.36 (1.13, 1.65)** | **1.48 (1.18, 1.86)** | 1.23 (0.90, 1.68) |

CMDs, cardio-metabolic diseases; CVDs, cardiovascular diseases; SHR, subdistribution hazard ratio; CHARLS, China Health and Retirement Longitudinal Study.

^a^ CMDs were defined as the presence of either CVDs or diabetes.

^b^ Adjusted for age, sex, marital status, education level, smoking status, alcohol consumption, and hypertension.

**Table S6.** Association of physical frailty (identified by FI) and cognitive impairment with CMD outcomes by Fine-Gray model (ELSA).

| **Group** | **CMDs**^a^ | **CVDs** | **Diabetes** |
| --- | --- | --- | --- |
|  | SHR (95%CI)^b^ | SHR (95%CI) | SHR (95%CI) |
| **Individual and combined effect** |  |  |  |
| Normal | Ref. | Ref. | Ref. |
| Frailty only | **1.64 (1.36, 1.98)** | **1.60 (1.30, 1.99)** | 1.44 (0.99, 2.09) |
| Cognitive impairment only | 1.06 (0.90, 1.25) | 1.01 (0.83, 1.23) | 1.24 (0.89, 1.73) |
| Cognitive frailty | **1.55 (1.18, 2.04)** | **1.65 (1.22, 2.24)** | 1.29 (0.74, 2.23) |
| **Combined effect vs. individual effect** |  |  |  |
| Cognitive frailty vs. frailty only | 0.95 (0.70, 1.28) | 1.03 (0.73, 1.45) | 0.89 (0.49, 1.63) |
| Cognitive frailty vs. cognitive impairment only | **1.47 (1.09, 1.97)** | **1.64 (1.17, 2.28)** | 1.04 (0.58, 1.85) |

CMDs, cardio-metabolic diseases; CVDs, cardiovascular diseases; SHR, subdistribution hazard ratio. ELSA, English Longitudinal Study of Ageing.

^a^ CMDs were defined as the presence of either CVDs or diabetes.

^b^ Adjusted for age, sex, marital status, education level, smoking status, alcohol consumption, and hypertension.

**Table S7.** Association of physical frailty (identified by PFP) and cognitive impairment with CMD outcomes in the combined CHARLS and ELSA cohorts.

| **Group** | **Cases, n (%)** | **CMDs**^a^ | | **CVDs** | | **Diabetes** | |
| --- | --- | --- | --- | --- | --- | --- | --- |
|  |  | Model 1^b^  HR (95%CI) | Model 2^c^  HR (95%CI) | Model 1  HR (95%CI) | Model 2  HR (95%CI) | Model 1  HR (95%CI) | Model 2  HR (95%CI) |
| **Individual and combined effect** |  |  |  |  |  |  |  |
| Normal | 9,472 (76.8%) | Ref. | Ref. | Ref. | Ref. | Ref. | Ref. |
| Frailty only | 306 (2.5%) | **1.73 (1.44, 2.09)** | **1.39 (1.14, 1.69)** | **1.72 (1.39, 2.12)** | **1.39 (1.12, 1.73)** | **1.66 (1.18, 2.33)** | 1.36 (0.96, 1.93) |
| Cognitive impairment only | 2,335 (18.9%) | **1.10 (1.01, 1.19)** | **1.10 (1.01, 1.20)** | 1.06 (0.97, 1.17) | 1.09 (0.98, 1.20) | 1.14 (0.99, 1.32) | 1.01 (0.94, 1.28) |
| Cognitive frailty | 218 (1.8%) | **1.54 (1.22, 1.94)** | **1.37 (1.06, 1.77)** | **1.51 (1.16, 1.96)** | **1.40 (1.05, 1.86)** | 1.51 (0.97, 2.33) | 1.34 (0.84, 2.15) |
| **Combined effect vs. individual effect** |  |  |  |  |  |  |  |
| Cognitive frailty vs. frailty only | NA | 0.89 (0.66, 1.19) | 0.99 (0.72, 1.35) | 0.88 (0.63, 1.22) | 1.01 (0.71, 1.43) | 0.91 (0.53, 1.56) | 0.99 (0.55, 1.75) |
| Cognitive frailty vs. cognitive impairment only | NA | **1.40 (1.10, 1.77)** | 1.24 (0.96, 1.61) | **1.42 (1.08, 1.86)** | 1.28 (0.96, 1.72) | 1.32 (0.84, 2.07) | 1.22 (0.75, 1.98) |

CMDs, cardio-metabolic diseases; CVDs, cardiovascular diseases; HR, hazard ratio; NA, not applicable.

^a^ CMDs were defined as the presence of either CVDs or diabetes.

^b^ Model 1 adjusted for age and gender.

^c^ Model 2 adjusted for age, sex, study region, marital status, education level, smoking status, alcohol consumption, and hypertension.

**Table S8.** Association of physical frailty (identified by PFP) and cognitive impairment with CMD outcomes (CHARLS).

| **Group** | **Cases, n (%)** | **CMDs**^a^ | | **CVDs** | | **Diabetes** | |
| --- | --- | --- | --- | --- | --- | --- | --- |
|  |  | Model 1^b^  HR (95%CI) | Model 2^c^  HR (95%CI) | Model 1  HR (95%CI) | Model 2  HR (95%CI) | Model 1  HR (95%CI) | Model 2  HR (95%CI) |
| **Individual and combined effect** |  |  |  |  |  |  |  |
| Normal | 5,915 (77.5%) | Ref. | Ref. | Ref. | Ref. | Ref. | Ref. |
| Frailty only | 227 (3.0%) | **1.26 (1.01, 1.57)** | **1.27 (1.02, 1.60)** | **1.33 (1.03, 1.71)** | **1.34 (1.04, 1.72)** | 1.22 (0.82, 1.83) | 1.23 (0.82, 1.84) |
| Cognitive impairment only | 1,364 (17.9%) | 0.98 (0.88, 1.09) | 1.02 (0.91, 1.13) | 0.96 (0.85, 1.09) | 1.00 (0.88, 1.14) | 0.99 (0.83, 1.18) | 1.02 (0.85, 1.23) |
| Cognitive frailty | 122 (1.6%) | 1.05 (0.75, 1.45) | 1.07 (0.77, 1.48) | 1.09 (0.75, 1.58) | 1.13 (0.78, 1.63) | 1.00 (0.55, 1.82) | 1.02 (0.56, 1.86) |
| **Combined effect vs. individual effect** |  |  |  |  |  |  |  |
| Cognitive frailty vs. frailty only | NA | 0.83 (0.56, 1.23) | 0.84 (0.57, 1.24) | 0.82 (0.53, 1.28) | 0.84 (0.54, 1.31) | 0.82 (0.40, 1.66) | 0.83 (0.41, 1.69) |
| Cognitive frailty vs. cognitive impairment only | NA | 1.07 (0.77, 1.50) | 1.05 (0.75, 1.47) | 1.14 (0.78, 1.67) | 1.12 (0.77, 1.65) | 1.01 (0.55, 1.87) | 1.00 (0.54, 1.84) |

CMDs, cardio-metabolic diseases; CVDs, cardiovascular diseases; HR, hazard ratio; NA, not applicable.

^a^ CMDs were defined as the presence of either CVDs or diabetes.

^b^ Model 1 adjusted for age and gender.

^c^ Model 2 adjusted for age, sex, study region, marital status, education level, smoking status, alcohol consumption, and hypertension.

**Table S9.** Association of physical frailty (identified by PFP) and cognitive impairment with CMD outcomes (ELSA).

| **Group** | **Cases, n (%)** | **CMDs**^a^ | | **CVDs** | | **Diabetes** | |
| --- | --- | --- | --- | --- | --- | --- | --- |
|  |  | Model 1^b^  HR (95%CI) | Model 2^c^  HR (95%CI) | Model 1  HR (95%CI) | Model 2  HR (95%CI) | Model 1  HR (95%CI) | Model 2  HR (95%CI) |
| **Individual and combined effect** |  |  |  |  |  |  |  |
| Normal | 3,557 (75.6%) | Ref. | Ref. | Ref. | Ref. | Ref. | Ref. |
| Frailty only | 79 (1.7%) | **2.66 (1.91, 3.70)** | **2.05 (1.40, 3.02)** | **2.31 (1.58, 3.37)** | **1.85 (1.19, 2.88)** | **2.56 (1.36, 4.84)** | 1.96 (0.95, 4.04) |
| Cognitive impairment only | 971 (20.7%) | **1.16 (1.02, 1.33)** | 1.13 (0.97, 1.32) | 1.10 (0.95, 1.29) | 1.10 (0.93, 1.31) | **1.33 (1.03, 1.72)** | 1.21 (0.90, 1.63) |
| Cognitive frailty | 96 (2.0%) | **2.20 (1.58, 3.08)** | **2.03 (1.35, 3.06)** | **2.03 (1.39, 2.96)** | **1.97 (1.25, 3.11)** | **2.50 (1.31, 4.76)** | **2.23 (1.03, 4.85)** |
| **Combined effect vs. individual effect** |  |  |  |  |  |  |  |
| Cognitive frailty vs. frailty only | NA | 0.83 (0.53, 1.31) | 0.99 (0.58, 1.70) | 0.88 (0.52, 1.48) | 1.06 (0.58, 1.97) | 0.98 (0.41, 2.35) | 1.14 (0.41, 3.14) |
| Cognitive frailty vs. cognitive impairment only | NA | **1.89 (1.34, 2.67)** | **1.80 (1.19, 2. 73)** | **1.84 (1.25, 2.72)** | **1.78 (1.12, 2.84)** | 1.88 (0.97, 3.64) | 1.85 (0.84, 4.06) |

CMDs, cardio-metabolic diseases; CVDs, cardiovascular diseases; HR, hazard ratio; NA, not applicable.

^a^ CMDs were defined as the presence of either CVDs or diabetes.

^b^ Model 1 adjusted for age and gender.

^c^ Model 2 adjusted for age, sex, study region, marital status, education level, smoking status, alcohol consumption, and hypertension.
